# Remote Modular Electronics for Wireless Magnetic Devices

**DOI:** 10.1002/advs.202101198

**Published:** 2021-07-10

**Authors:** Mustafa Boyvat, Metin Sitti

**Affiliations:** ^1^ Physical Intelligence Department Max Planck Institute for Intelligent Systems Stuttgart 70569 Germany; ^2^ School of Medicine and College of Engineering Koç University Istanbul 34450 Turkey; ^3^ Institute for Biomedical Engineering ETH Zurich Zurich 8092 Switzerland

**Keywords:** magnetic robots, modular devices, reconfigurable devices, wireless devices, wireless power transfer

## Abstract

Small‐scale wireless magnetic robots and devices offer an effective solution to operations in hard‐to‐reach and high‐risk enclosed places, such as inside the human body, nuclear plants, and vehicle infrastructure. In order to obtain functionalities beyond the capability of magnetic forces and torques exerted on magnetic materials used in these robotic devices, electronics need to be also integrated into them. However, their capabilities and power sources are still very limited compared to their larger‐scale counterparts due to their much smaller sizes. Here, groups of milli/centimeter‐scale wireless magnetic modules are shown to enable on‐site electronic circuit construction and operation of highly demanding wireless electrical devices with no batteries, that is, with wireless power. Moreover, the mobility of the modular components brings remote modification and reconfiguration capabilities. When these small‐scale robotic modules are remotely assembled into specific geometries, they can achieve, if not impossible, challenging electrical tasks for individual modules. Using such a method, several wireless and battery‐free robotic devices are demonstrated using milli/centimeter‐scale robotic modules, such as a wireless circuit to power light‐emitting diodes with lower external fields, a device to actuate relatively high force‐output shape memory alloy actuators, and a wireless force sensor, all of which can be modified on‐site.

## Graphical abstract

Small‐scale wireless magnetic robots have no or limited electrical functionalities due to their limited space and battery capacity. A modular approach with wireless radio frequency power transfer is demonstrated to boost the electrical capabilities of such robots at the milli/centimeter scale. Such an approach enables remote assembly and operation of reconfigurable wireless electrical devices on wireless magnetic robots achieving various tasks.

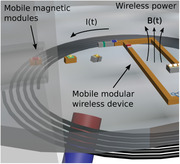

## 1 Introduction

Small‐scale wireless robots and devices are indispensable to perform operations in hard‐to‐reach and high‐risk enclosed places, such as the inner parts of the human body, in nuclear and industrial plants, and other infrastructure inspection or repair scenarios where external wires are not desired.^[^ ^1^ ^]^ Using magnetic forces and torques exerted on magnetic bodies is a promising method to move small‐scale robots as external magnetic fields and gradients can provide the necessary actuation for locomotion and robotic functions in a fast, precise, and controllable way, and they can pass through the vast majority of materials without a significant perturbation when their frequency is low.^[^ ^1^, ^2^, ^3^, ^4^, ^5^ ^]^ However, they do not offer the complexity of large‐scale robots, which benefit from onboard integrated electronics. To increase the functionality of small‐scale mobile robots towards their larger‐scale counterparts, electronic components can also be integrated to them. Yet, conventional methods to provide electrical power for long durations, such as using batteries, start to become problematic at smaller length scales due to the volumetric scaling laws. Electromagnetic wireless power transmission technique offers a promising solution to the power problem of functional small‐scale robots for longer duration operations.^[^ ^2^, ^3^, ^4^, ^5^, ^6^, ^7^, ^8^, ^9^, ^10^ ^]^ Nevertheless, in general, applied external magnetic fields cannot be increased arbitrarily and the delivered power amount is limited by the size of the wireless receiver, thus, the size of the robotic devices.^[^ ^9^, ^10^, ^11^, ^12^ ^]^

**Figure 1:**
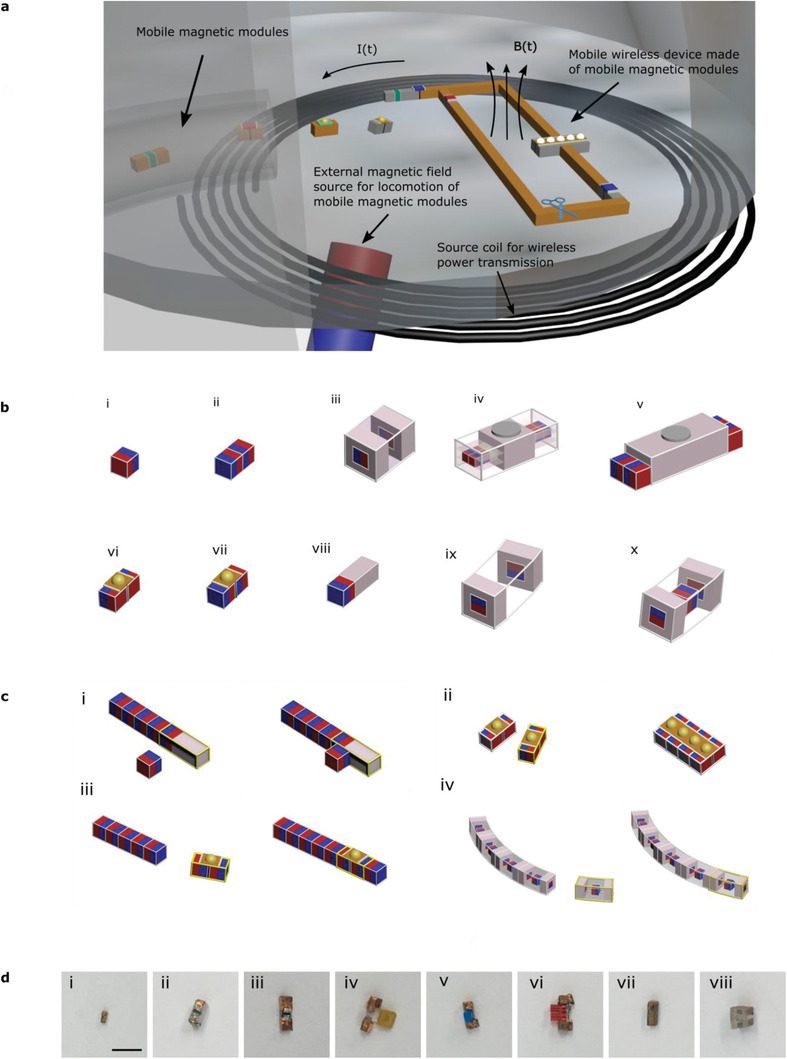
Schematic concept of assembly of diverse magnetic modules into operational wireless electronics for untethered robotic devices. a) Illustration of a wireless power system and mobile magnetic modules forming a circuit with wireless power transfer capability. The larger coil produces a time‐varying magnetic field and the built circuit receives power through this field. b) Examples of possible magnetic modules. Magnets are shown by a pair of blue and red blocks, corresponding to the two poles of magnets. The modules in (i) and (ii) are single magnet and pack of magnets, respectively. (iii) A connection module with a preferential connection orientation and can be converted to a component module by loading with a component. (iv,v) Examples of advanced electronic component modules used for series and parallel receiver coil connections. (vi–viii) Examples of electronic component modules with different magnet orientations and an L connector. (ix) Module for circular geometries and (x) is the improved version of ix with a magnet at the center. c) Connection examples: (i) is an L connection and becomes a T connection when attached from two sides. (ii) Example parallel connection of the component modules. (iii) Series connection of a component module. (iv) Circular connection. d) Examples of experimental modules used in this study. Scale bar: 1 cm. (i) Connection module similar to Figure 1b(ii). (ii) Receiver coil module for series connection similar to Figure 1b(iv). (iii) Receiver coil module for parallel connection similar to Figure 1b(v). (iv–vi) Electronic component modules for series and parallel connections similar to Figure 1b(vi,vii). (vii) L connection module similar to Figure 1b(viii). (viii) Circular connection module similar to Figure 1b(x).

Having robotic modules in groups can increase functionalities and there are a large number of examples of relatively intelligent robot groups as well as less autonomous modules achieving complex tasks from coordinated motion to collective transport when they act altogether.^[^ ^13^, ^14^, ^15^, ^16^, ^17^ ^]^ Tasks achieved through groups do not stay only in the mechanical domain but also extend to electronics‐related tasks, such as self‐assembly of electronic circuits.^[^ ^18^, ^19^ ^]^ Magnetic robots have also been studied for group tasks, such as assembly, but most of the work at milli/centimeter and smaller scales do not focus on enhancing the electrical capacity beyond individual modules, and enhancement of electrical operations by groups are mostly seen in magnetic modules of larger scales, where the energy storage is a relatively less significant problem or in cases where electrical power is provided by external wires.^[^ ^20^, ^21^, ^22^, ^23^, ^24^, ^25^, ^26^, ^27^, ^28^, ^29^, ^30^, ^31^, ^32^, ^33^, ^34^, ^35^, ^36^ ^]^ For example, it has been demonstrated that small magnetic modules can serve as connection conductors for an existing electronic circuit or they can be used in the placement of components for intra‐modular or module‐to‐substrate assembly.^[^ ^37^, ^38^, ^39^ ^]^ Nevertheless, the question of obtaining long‐duration powers and functionalities exceeding single module capacities for the completed electronic circuits still remains when the completed circuits are desired to be wireless and mobile. However, assembling small‐scale magnetic robotic modules remotely into mobile untethered electrical devices with a constant and enhanced wireless power source offers a significant potential to have capabilities at the level of larger‐scale electronic robotic devices in hard‐to‐reach places. Here, we demonstrate operational mobile electronic wireless robotic devices enabled by groups of milli/centimeter‐scale mobile magnetic modules (**Figure** **1**a). By assembling into functional mobile electronic circuits, which can receive wireless power continuously, these modules can achieve relatively challenging tasks going beyond individual modules. Moreover, they can relax the requirements, such as the necessary field strengths used in magnetic wireless power transfer. The magnetic modules are designed to be connected either sequentially, which gives a certain level of freedom in the assembled circuit topologies, or are designed to form pre‐determined preferential geometries even if they are not connected sequentially. The delivery of modules to the operation site is realized by manipulating them by external permanent magnets, but other delivery methods such as gravity‐based or flow‐based motions can also be used when modules are designed for a pre‐determined geometry. As the approach is based on modularity and magnetic attachment forces are breakable, the devices are also reconfigurable up to a certain level.

We demonstrate the proposed method by using mm‐cm size magnetic mobile conductors and components to make different types of circuits in 2D: a) a light source with its receiver circuit and light‐emitting diodes (LEDs) is built to show the concept, and such a circuit can be changed on‐site by component replacement or extending the electronic circuit; b) a strong shape memory alloy (SMA) actuator is powered and used to move a heavy load with the possibility to alter its operation frequency on site, which is relatively very challenging for the power levels that can be provided by a single mm‐cm scale unit; c) a resonance‐based wireless force sensor with a tunable resonance is shown as an example sensor device application. As the demonstrations show, the proposed method can enable the operation of high‐power electronic devices, which are delivered through robotic modules at the same delivery procedure or placed/implanted before the modules are delivered. As wireless power removes the necessity for batteries, long‐duration operations can become possible. It can also help devices with batteries by wirelessly charging the batteries. Its modular nature can facilitate flexibility, reconfigurability, and optimization of module sizes for individual deliveries, such as keeping delivery package sizes at a minimum when they have to pass through narrow routes and delivering several modules together in suitable cases to speed up the process. Moreover, it can provide a certain level of on‐site modification possibility, which means that the method does not only offer building‐specific circuits for any given case but also changing or reconfiguring them when the application requirements change. It is foreseen that the proposed method can extend the spectrum of potential applications of miniature wireless robots significantly by enabling enhanced and reconfigurable onboard electrical capabilities.

## 2 Module Design and Geometry Formation

Modules to form wireless circuits, devices, or robots can be categorized mainly as connector modules and component modules. Connector modules are used to make conductive connections and act as wires of circuits. They can be as simple as single magnets coated by a conductive material or can be a combination of magnets and other materials. As the formation geometry is important, we have various connector module types to be able to build different geometries as seen in Figure 1b. By having modules for straight connections, L connectors, and T connectors, one can form a large number of different circuit geometries with different sizes. Possible complications related to non‐unique connections can be solved through sequential assembly strategies. Modules can also be designed in certain shapes to form predefined geometries which significantly reduces the effort during the assembly process.

The L connectors can be designed in various forms. A simplistic way for an L connector, which extends a cubic module only in 1D, can be seen in Figure 1c(i). Extending the modules only in 1D can help the modules pass through smaller channels, for example, esophagus, intestines, and tubular regions in industrial plants. An approach to realize the L connector with a 1D extension is to combine a single magnet with a dummy non‐magnetic part as seen in Figure 1b(viii). Another possible idea is to combine two magnets whose magnetizations are perpendicular to each other, but it can require more effort in assembly due to an additional magnet with a preferential connection direction. The rod module made of a magnet and a dummy part would have a relatively weaker attachment at the side, but after making its straight connection, the second connection at the side can be formed in all directions (Figure 1c(i)). If the size is not a major concern, two magnets and one dummy cube can also be combined in an L shape to form an L‐connector module. Even though this approach for the L connector makes the module larger in 2D, it can be advantageous for its stronger connections at its ends. However, it also requires careful attachment as its L connection has a preferential direction. T connectors can be obtained by using the rod‐type L connector mentioned above. This would make one strong connection at the magnet end and two (or even more if we consider 3D) weaker connections at sides (Figure 1c(i)). Similar to the L connector, T connectors in T shape can also be an option. Component modules are the modules that carry electronic components with connections ready for magnetic attachment. Depending on the number of required connections and desired connection directions, they can have different structures (see Figure 1b,c). Examples of experimental modules used for the demonstrations in this work can be seen in Figure 1d.

**Figure 2:**
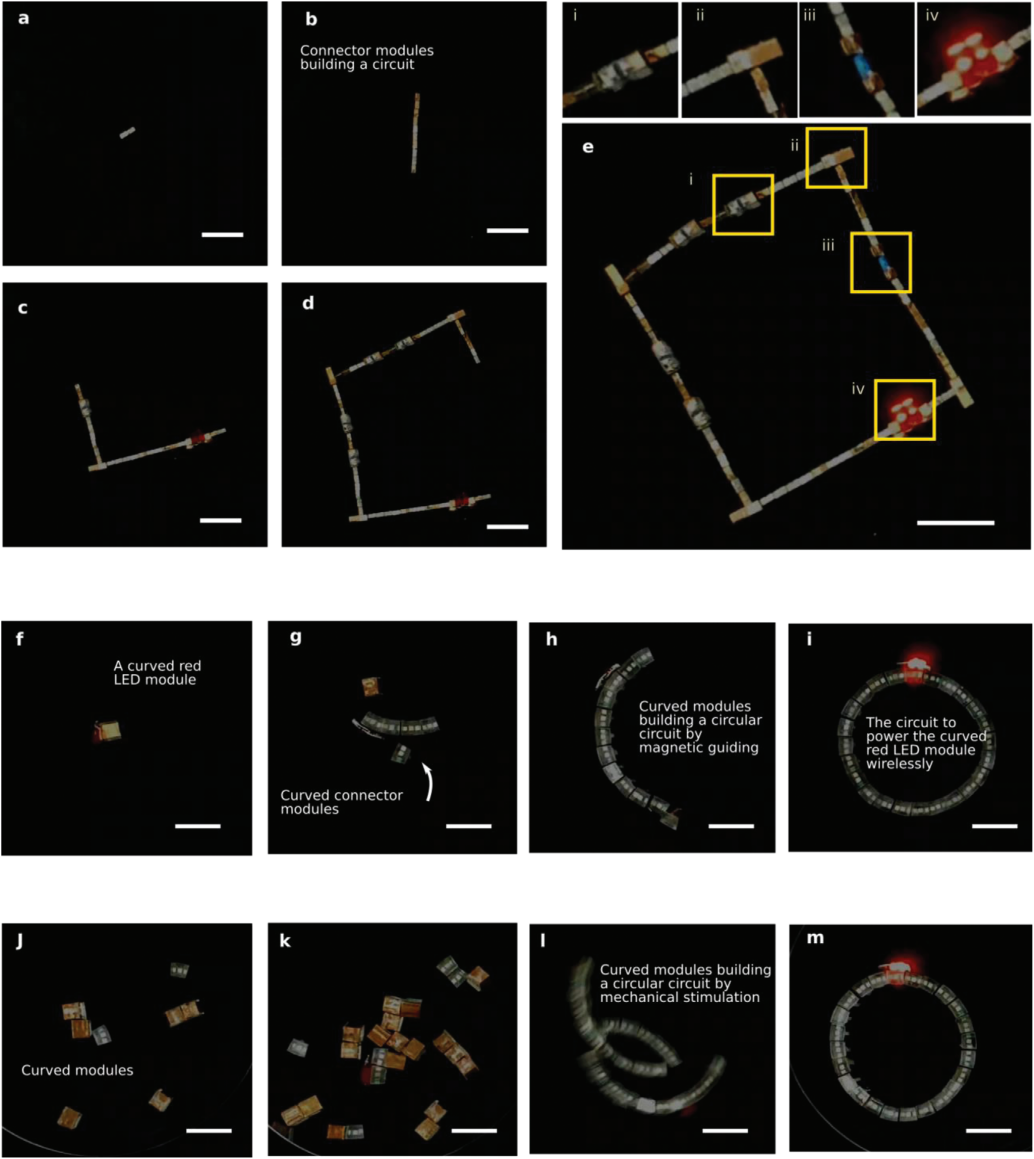
Various approaches to building electronic circuits with mobile magnetic modules and wireless power transfer capability. a–e) demonstrate how a square‐shaped circuit can be built by carrying the modules with a magnet sequentially. The built circuit has four receiver coil modules (i), four L‐connector modules (ii), one capacitor module (iii), and an LED module (iv). Linear connection modules are made of two 2 × 2 × 2 mm^3^ cubic NdFeB magnets covered with copper tape. The L connector is a rod‐type connector with a 3 × 3 × 3 mm^3^ cubic magnet. The modules need to be brought sequentially to form the desired shape. The power of the circuit is provided by a coil underneath (not shown, see Experimental Section for the details). f–i) show how a circle‐shaped circuit can be built by bringing modules one‐by‐one by a magnet to the target. j–m) demonstrate that the circuit can be formed by mechanical stimulation as well without any module order concern. Again, the power for the circular circuit is provided by the coil underneath (not shown). All scale bars are 2 cm.

An example of building a square‐like wireless circuit using 59 modules can be seen in **Figure** **2**a–e and Video S1, Supporting Information. The circuit is made of four receiver coil modules (Figure 2e(i), similar to the module in Figure 1b(iv)), four rod‐type L‐connector modules (Figure 2e(ii), similar to the module in Figure 1b(viii)), one capacitor module (Figure 2e(iii)), one LED module (Figure 2e(iv)), and 49 conductor modules which were delivered mostly two by two. The modules are moved by larger magnets to the target area sequentially. The connection of the conductor modules to each other to take a line shape is relatively straightforward: they are brought one by one and they are connected either by moving the incoming module or the part in the target area. When they are close enough, they attract each other when the larger magnet is kept far enough (or kept in a direction to encourage the desired connection). L connectors are brought in a way to connect in a line shape from their magnet end (Figure 1c(i)). The following connector module is brought from the desired L connection direction. After the connection of the following connector module, the corner is constructed and the simple linear connections follow again.

The capacitor and LED modules are similar to the modules shown in Figure 1b(vii), therefore, very similar to connector modules in the connection process. When the circuit gets larger, it is also possible to control this large part using a large magnet from a further point of the part, that is, from a point where the large magnet can stay far from the connection point. This can be used to move the large part rather than the small incoming module in a way to minimize the interaction of the incoming module with the large magnet. When the larger part gets large and too heavy, using two larger magnets can also help. An important point in the connection is adding the receiver coils in the correct orientation, that is, all should have the same sign of the induced voltage at a time to enhance the total induced voltage. Therefore, their magnets are made in two groups: one group at the outer parts for simple linear connection, and the other group is in a perpendicular direction to keep the orientation of the coil in the desired direction, as seen in Figure 1b(iv). During assembly, moving the larger part from a further point of it towards the receiver coil module sometimes becomes rather helpful as this can help keep the orientation of the receiver coil module more stable. Towards the end, using two magnets can help prevent the circuit from forming a loop too early by keeping one of the “legs” away from the connection tip. Another solution to too‐early closing up is to form the last closing piece separately before the open ends of the circuit are close enough to be able to connect and bring this piece to the main circuit instead of bringing those modules one by one. How larger magnets can be used to form such circuits is shown in Video S2, Supporting Information.

It is also possible to design modules in a way that requires less effort during the assembly process, such as curved unit modules to connect in a circular shape as seen in Figure 1b(ix,x),c(iv). By embedding magnets inside non‐magnetic holders of certain shapes, these modules can be designed to have preferential formation geometries. It means that there is less freedom in the formation geometry after preparing modules but it can offer assembly of modules in the designed orientations and they can even be delivered in random orders. Figure 2f–i and Video S3, Supporting Information, show how a circular geometry can be built by bringing modules one by one using an additional magnet. In this case, it is rather straightforward to form the shape. The modules are similar to the module design in Figure 1b(x); two magnets are enclosed with a non‐magnetic material at the tips to have a single connection direction and they are placed with opposite directions to have a circular connection whichever tips come together, and another magnet is put at the center to enhance the net magnetization of the module otherwise consisting of two magnets having nearly opposing magnetic moments. Figure 2j–m and Videos S4 and S5, Supporting Information, show that the same modules can also be connected by mechanical vibration with no sequential order issue to form the desired 2D geometry.

## 3 Modification or Reconfiguration of the Formed Circuits

**Figure 3:**
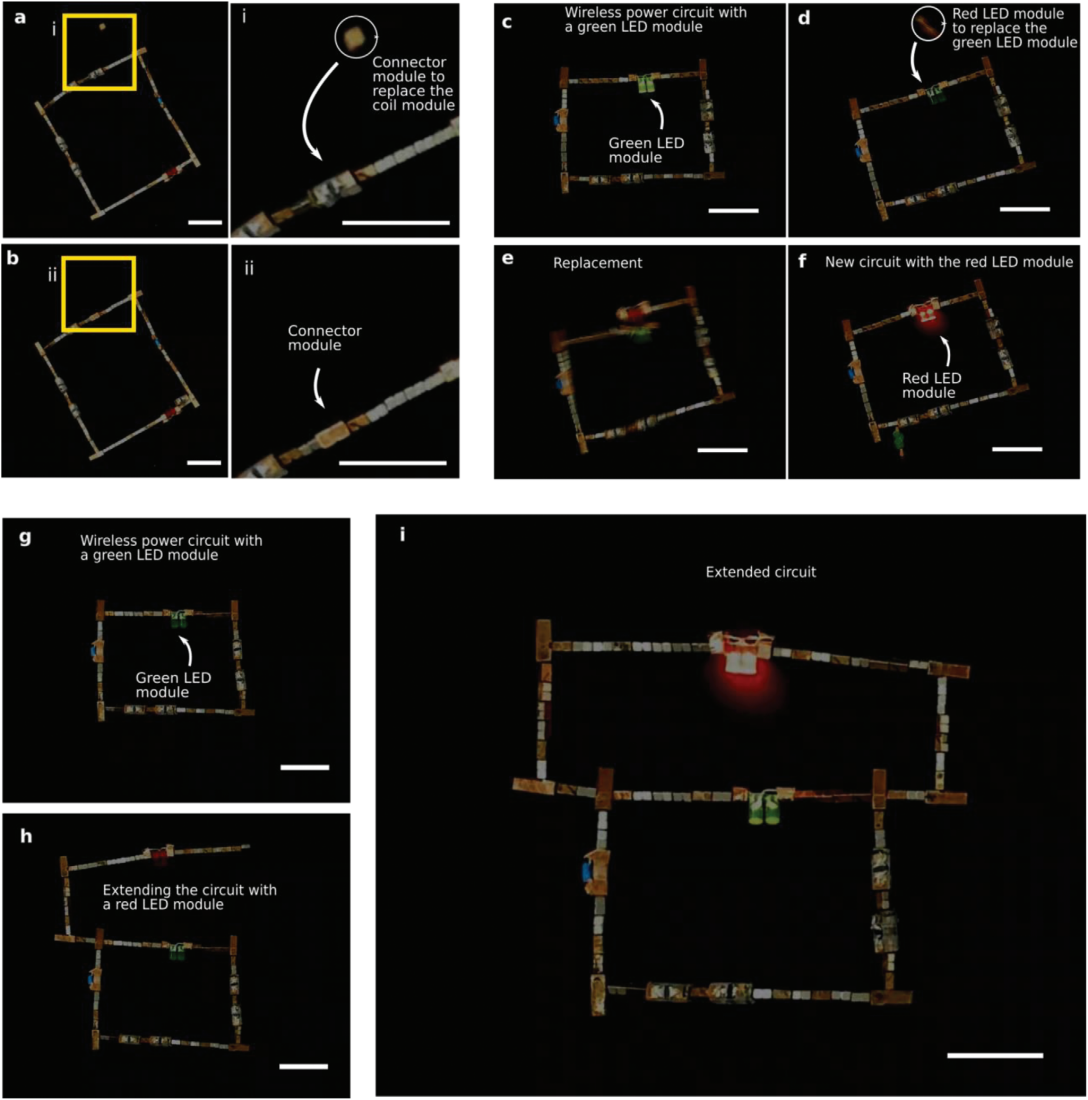
On‐site modification or reconfiguration of wireless circuits. a,b) Modification of the circuit in Figure 2e by replacing a receiver coil with a connector module made of two 3 × 3 × 3 mm^3^ magnets. The connector is brought close to the component to be replaced by a magnet and the receiver coil module is pushed away as its connections are relatively weak. The new circuit has less receiver coils and a different resonance frequency. c–f) Replacement of a green LED module by a red LED module as a demonstration of function change. The green LED module is built by weaker magnetic connectors and red LED module replaces it when the red LED module is brought near the green LED module by a magnet. g–i) Modification by geometry change. By extending the circuit, a red LED is connected in parallel to a green LED. All scale bars are 2 cm.

The circuits formed can be changed or reconfigured after initial assembly by component replacement or by extending the circuit with new modules. **Figure** **3**a,b show how a stronger connector module replaces a receiver coil module (see also Video S6, Supporting Information). This happens because the connection of the receiver coil module is weak enough to be broken by stronger magnets. This particular modification would change the total induced voltage and the resonance frequency of the circuit. Figure 3c–f and Video S6, Supporting Information, show how a green LED is replaced by a red LED to demonstrate a functional change in the circuit. In Figure 3g–i and in Video S7, Supporting Information, it is exemplified how a circuit can be changed to a different circuit by adding new geometries by adding a red LED to a circuit which already has a green LED.

## 4 Wireless Power Transmission

Electromagnetic wireless power transmission is a promising powering method for small‐scale devices with limited battery capacity and used at low frequencies as well as high frequencies.^[^ ^7^, ^8^, ^9^, ^11^, ^12^, ^40^, ^41^, ^42^, ^43^, ^44^, ^45^, ^46^, ^47^ ^]^ When the frequency of the field is low and the medium does not cause a significantly high perturbation, it is possible to obtain large operation spaces having almost uniform fields as seen in several previous small biomedical robot studies.^[^ ^40^, ^41^, ^42^, ^43^ ^]^ However, when the size of the receiver coil is small, it is challenging to transmit high power due to the factors such as the small area for magnetic coupling and the limitations on the applied magnetic fields, for example, the International Commission on Non‐Ionizing Radiation Protection (ICNIRP) regulations given for the human body.^[^ ^12^, ^40^, ^41^, ^42^, ^43^, ^48^ ^]^

The delivered power which can be delivered to a load by a receiver, which is run at resonance by adding a capacitor and loaded with a matched resistance, can be calculated by $P_{\max}=V_{\mathrm{ind}}^{2}/\left(2R\right)$, where *V*_ind_ is the induced voltage and *R* is the resistance of the resonator without the load for given external field strength.^[^ ^46^ ^]^ To increase the power capacity further using multiple receivers, a possible approach is to connect them in series assuming that they are in a uniform field and both total induced voltage and total resistance increase as: *V*_tot,ind_ = *n* · *V*_ind_ and *R*_tot_ = *n* · *R* (neglecting the connection resistances), and power capacity increases to *P*_tot,max_ = *n* · *P*_max_, where *n* is the number of receivers. Therefore, the power capacity of the system would increase *n* times compared to the single receiver capacity, if the dominant resistance in the circuit is the receiver coil resistance. Or, equivalently, the magnetic field strength *B* required to achieve a certain power capacity would decrease to $B/\sqrt{n}$, and the undesired power losses *P*_tr,loss_ in the source and the surrounding medium, for example, the power loss in the human body if it is delivered to the inner human body, would decrease to *P*_tr,loss_/*n*, with a linearity assumption. In some cases, it is possible to have hard component limitations given by the manufacturers and even increasing the field is not helpful to increase the received power capacity anymore. In such cases where the limitation is not the applied field but the limitations of the components, using multiple elements can increase the limit of the circuit by distributing the electrical tension on components. The powering of multiple receivers can also be supported by a properly arranged formation geometry, that is, by forming large loops, to increase the coupling to the external magnetic field.

The calculations above are for linear loads driven by alternating currents (AC). When there is a need for direct current (DC) voltages and AC to DC conversion, or there are certain threshold voltages to be reached, the advantage is even more vital as operating at low AC voltages is relatively more challenging.^[^ ^12^ ^]^ For example, if the circuit is assembled to drive LEDs, normally a certain threshold voltage is needed, and by using *n* elements, the minimum field *B*_min_ required to reach this threshold may decrease almost to *B*_min_/*n*, which means the undesired losses *P*_tr,loss_ at this minimum required field may decrease to almost *P*_tr,loss_/*n* ^2^.

The received power capacity in given field strength can also be increased by connecting multiple receivers in parallel. Assuming that the field is uniform, the induced voltage in each receiver would be the same but the resistance *R* would decrease to *R*/*n*, neglecting the resistance coming from the connections. Therefore, the power capacity again would increase to *n* · *P*_max_. This configuration is more suitable for high‐ current low‐voltage cases as it mostly increases the current. Again, it can be used to provide a higher current than the limit of individual receivers. Moreover, there is a contribution from the large loop in the order of a contribution of two receiver coils. However, the mentioned contribution of the loop compared to receiver coils is specific to this particular circuit and can change simply by using receiver coils with a different number of turns. In Figure 2e, the minimum power used to see a light in the LEDs is calculated to be several tens of times less compared to a single receiver coil (the field to induce the same voltage in a single receiver coil is found to be 6.4 times the field needed in the formed circuit). The details of the wireless power transmission can be seen in Experimental Section.

## 5 Wireless Actuation of a Shape Memory Alloy Actuator and High‐Temperature Heater

**Figure 4:**
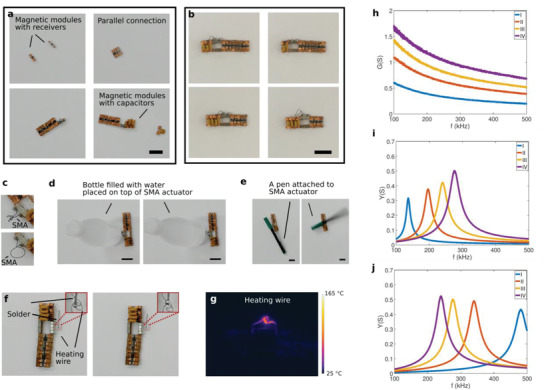
Wireless actuation of a high‐force output SMA actuator and wireless heating. a) A resonance circuit with an SMA actuator is built by multiple modules. b) Various combinations of inductors and capacitors can be made to adjust the size, resonance frequency, and resistance in the circuit on‐site. c) Actuation of the SMA actuator, which is beyond the capacity of a single module. d) SMA actuator placed under a 200 mL bottle of water moving it up by wireless power. e) SMA actuator rotating a pen attached to it using wireless power. f) Melting a piece of solder wire in several seconds. The melting temperature of the solder is given in the 183–190 °C range by the manufacturer. g) Temperature profile during a heating process. h) Conductance of receiver coil modules connected in parallel: 2 (I), 4 (II), 6 (III), and 8 (IV) coils. i) Admittance as a function of frequency for different number of receiver coils: 2 (I), 4 (II), 6 (III), and 8 (IV). When the number of receiver coils changes, the resonance frequency also changes. j) Admittance as a function of frequency for 1 (I), 2 (II), 3 (III), and 4 (IV) capacitor modules. The resonance frequency can be adjusted by using a different number of capacitor modules. All scale bars are 2 cm.

A wireless receiver circuit to drive a high force‐output SMA actuator is shown to demonstrate how multiple receiver coils can combine their power to go beyond their limits. The used SMA actuator has a diameter of 300 µm, a resistance of about 1 Ω, and requires a current of about 1–1.5 A in the air. The individual receiver coils have a current limit of 0.26 A (root‐mean‐square (RMS)) according to their datasheet. By coming together, they can directly actuate this demanding actuator. How they can come together to form a parallel connection can be seen in **Figure** **4**a and in Video S8, Supporting Information. Their design is similar to the module shown in Figure 1b(v). Unlike the series connection of receiver coils in Figure 2a, the connection orientation of the receiver coils here does not require attention as their connection tips also change in the case of orientation changes, that is, they have the same sign of the induced voltage in both connection orientations.

The built parallel receiver coil group is then connected to the SMA actuator and the capacitors as seen in Figure 4a, and the circuit is closed with a conductor module similar to the circuits shown in Figure 4b. How a strong SMA actuator in such circuit is actuated is demonstrated in Figure 4c (states before actuation and after actuation) as well as Video S8, Supporting Information. Figures 4d,e and Video S8, Supporting Information, demonstrate the high force‐output of the actuator by pushing up a 200 mL bottle filled with water and rotating a pen attached to it. Figure 4f shows a high‐temperature heater made using a resistive wire, which can melt a piece of solder wire in several seconds, and Figure 4g shows the temperature map of the heater during the heating process. The method also offers a certain level of on‐site modification flexibility. Figure 4b shows examples of various resonance receiver circuits with a different number of receiver coils and capacitors. This enables the possibility of changing the characteristics of the receiver group and also the resonance frequency of the circuit even after delivery. Figure 4h shows how the effective conductance increases with the increasing number of coils. Figure 4i shows the admittance of the receiver circuit with respect to the frequency and the effect of increasing the number of receiver coils on the admittance curve while keeping the capacitance the same. When more coils are added, the inductance and the effective series resistance of the receiver coil group are reduced and the resonance frequency increases as seen in Figure 4i. Figure 4j shows how the resonance frequency changes by changing the capacitance while keeping the receiver coil group the same.

## 6 Resonance‐Based Wireless Force Detector/Sensor with On‐Site Modification Capability

**Figure 5:**
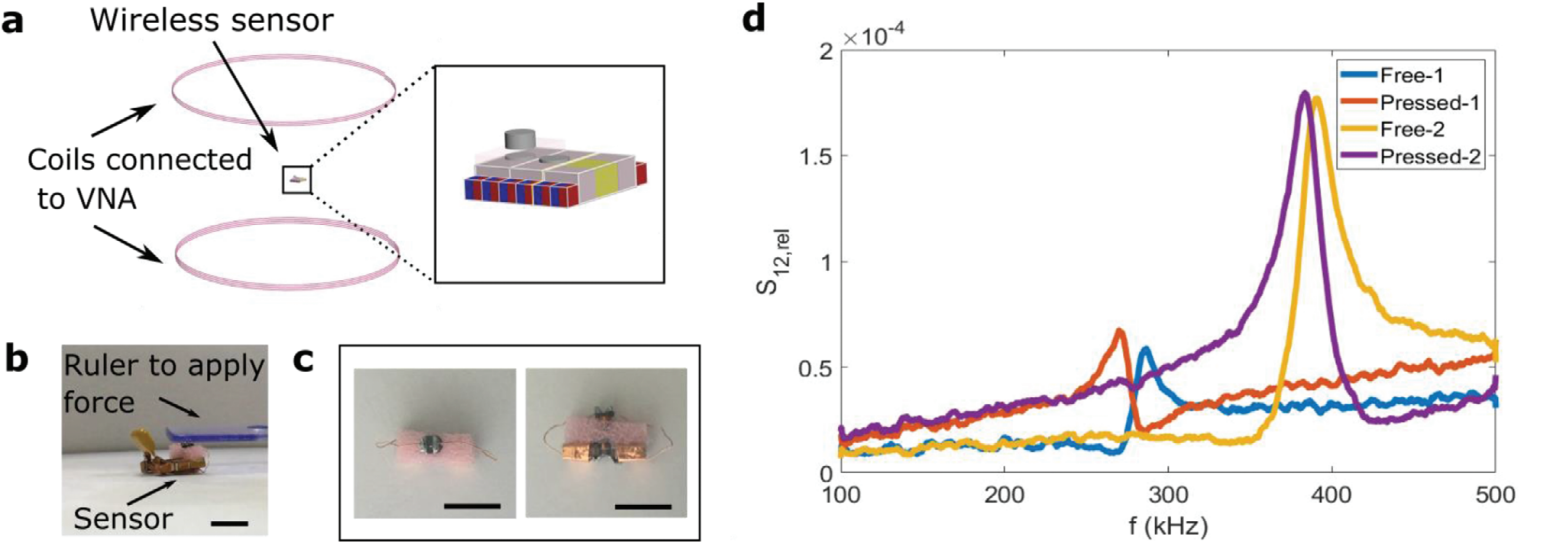
A resonance‐based wireless detector/sensor demonstration. a) Wireless force detecting method. When the mutual coupling between two coils connected in parallel in the sensor changes, the resonance frequency of the circuit they are connected in also changes. This change is detected by the change in the scattering parameter S_12_ of two external coils connected to a VNA (vector network analyzer). b) Photo of the wireless force sensor device. A ruler is used to apply a contact force/pressure. c) Photo of the force sensor module separately. Two coils are connected in parallel and a sponge is placed in between to serve as a spring. d) The change of relative S_12_ with the application of a force for two different resonance frequencies. The resonance frequency shifts when the sensor is pressed. Similar to the SMA circuit shown in Figure 4, the resonance frequency the sensor operates around can be changed by using a different number of coils and capacitors as shown before. Scale bars are 1 cm.

Another example of the abilities of the proposed method is demonstrated by a simple resonance‐based wireless force detector (sensor) circuit (**Figure** **5**a–c), which can be modified on‐site. The components of the detector (sensor) circuit are very similar to the SMA actuation circuit. It is composed of only receiver coil module(s), capacitor module(s), and the sensor module. The sensor module is a pair of coils connected in parallel and a sponge is placed in between them (Figure 5b,c). When the distance between them changes due to forces, the effective inductance would change. As it is connected to a resonance circuit, this changes the resonance frequency of the circuit. The sensor circuit is placed between two large coils as shown in Figure 5a and the change in the scattering parameter S_12_ of these two coupled coils is measured to detect any shifts in the resonance for the force sensing. By modifying the number of receiver coils and the capacitors, the features of the sensor can be changed for various potential purposes, for example, to control the magnitude or frequency changes caused by exerted forces (Figure 5d). This demonstration is intended for proof of concept only and is open to modification and improvement by using other sensing and detection methods and materials.

## 7 Discussion

Using groups of small‐scale modules can take the untethered magnetic robot operations enabled by wireless power and remote electronics to a higher level. By mobilizing the components and wire connections of a wireless circuit, new wireless miniature robotic operations in enclosed spaces can become possible. By having groups of modules assembling into wireless power‐equipped electronics, it is possible to receive high electrical power levels which are difficult, if not impossible, to achieve by individual robots. Moreover, the robotic modules can build circuits with various geometries, which can be important for circuits consisting of relatively large components requiring separate delivery or for elements whose performances depend on their geometries, such as antennas. Furthermore, on‐site modification is also possible.

The formed circuits can have flexibility in their geometry by sending designed modules in specific orders to the operation site as shown in Figure 2a–e. It is also possible to design modules for one geometry and remove the necessity of sending them in specific orders as in Figure 2f–m. These can also be combined by keeping certain parts less flexible but easier to assemble and certain parts to be sent at the right time to be able to control the geometry of the circuit to have a certain level of flexibility. The demonstrations are in 2D, but, by designing some of the modules to make out‐of‐plane connections, it can be possible to build 3D circuits. An important factor in this is the fact that when the assembly is conducted in 3D, the mechanical support profile given by the surfaces they are built on also needs to be considered for integrity as well as the effect of the external magnet on the integrity. Another point that can be studied in the future is the disassembly of the devices, which can be realized primitively by pulling the assembled devices to narrow passages to let the magnetic bonds break at the current state of the study.

The proposed method can be used for various electronic and robotic components. Miniature SMA actuators (which can be used in a large number of robotic devices), light sources, sensors, and powerful heaters are a few examples of such uses only. Even though the working distance of the receivers from the transmitters and the module designs can still be further optimized for their size scale and performance, it can be foreseen that the proposed method can enable a large number of wireless miniature robot and device applications, which are not possible at the moment.

## 8 Experimental Section

### Materials

Magnets used in the connector modules were nickel‐coated 2 × 2 × 2 mm^3^ and 3 × 3 × 3 mm^3^ cubic NdFeB magnets. The modules were covered with a copper tape for higher robustness and conductivity (Advance tapes, AT526 35 Micron Copper Foil Shielding Tape). The SMA actuator (Nitinol) used has a diameter of 300 µm. The solder used in the heating demonstration was Stanol HS10 with a given melting temperature of 183–190 °C. The device used for temperature profile acquisition was a FLIR ETS3 infra‐red (IR) camera. The receiver coils used in the demonstrations were 100 µH, 3 mm diameter, SDR0302‐101KL inductors from Bourns. The maximum AC given in the datasheet was 260 mA. The capacitor used in the LED demos was a 1 nF capacitor and the ones used in the SMA actuation and wireless sensor were 10 nF capacitors.

### Wireless Power Transmission

In the wireless radio frequency (RF) power transmission system, a resonator made of a spiral coil (square shaped with rounded corners) with 16 turns and capacitors was coupled to a loop connected to an RF amplifier (Broadband Amplifier, Rohde & Schwarz BBA150‐A160BC160) fed by a function generator (RIGOL DSG3060). The size of the loops was approximately 20 cm x 20 cm. In the demonstration of SMA actuation, the source coil has a diameter of about 10 cm and about 24 turns. The wireless power experiments shown were performed at small transmitter‐receiver distances, that is, on the order of 1 cm. The magnetic field of the source coils was found by placing a 4‐turn receiver coil with a 3 cm diameter at the center. An average value for the magnitude of magnetic field flux density *B* was calculated by Faraday's law as:

(1) $$V=2\pi f\cdot B_{\mathrm{avg}}\cdot A\cdot N$$

where *V* is the measured induced voltage, *f* is the frequency of the applied field, *B*_avg_ is the average magnetic flux density, *A* is the area of the receiver loop, and *N* is the number of turns.

In the LED experiments, the frequency of the applied magnetic field was about 249 kHz and the RMS value of the magnetic flux density *B*_avg_, at the receiver position around the level LEDs started to shine for the case of the circuit in Figure 2e, was approximately 82 μT RMS. Power delivered by the RF amplifier to produce the field around was around 1 W, most of which was wasted in the transmitter coil and cables. These values can be decreased even further by optimization of the components used.

In the case of the smaller source coil used in the SMA actuation, the frequency was around 243 kHz. The field values used were calculated to be on the order of 700 μT RMS. In the high‐temperature heating experiment, the heating element was a resistive load (wire) with a DC resistance of about 1.5 Ω. The field values used were around 920–930 μT RMS, which can be reduced by using shorter resistive heating wires.

### Wireless Sensing

For wireless sensing of the resonances, two coils of 14 cm diameter were placed axially aligned. One of them has 3 turns and the other one 4 turns. They were connected to a Network Analyzer (Keysight E5061B) and the sensor module was placed in between these large coils, keeping the receiver coils also axially aligned with the larger coils to have high coupling. The distance of the sensor from each of the coils was on the order of 5 cm. First, S_12_ was measured with no sensor module in between and used as a reference for the force sensing measurements as force measurement was based on the change of relative S_12_.

### Statistical Analysis

The data were represented as single instances after experiments were done to observe the main results. The data presented was the data taken from the equipment and any important data processing was performed by the equipment.

## Conflict of Interest

The authors declare no conflict of interest.

## Author Contributions

M.B. proposed the concept. M.B. and M.S. designed the research. M.B. designed and performed the experiments, and analyzed the data. M.B. and M.S. wrote the manuscript.

## Supporting information

Supporting Information

Supporting Video 1

Supporting Video 2

Supporting Video 3

Supporting Video 4

Supporting Video 5

Supporting Video 6

Supporting Video 7

Supporting Video 8

Supporting Video 9

## Data Availability

All data needed to evaluate the conclusions in the paper are present in the paper or the Supporting Information.
