# Supplementary material for: Remote Modular Electronics for Wireless Magnetic Devices
Source: Adv Sci (Weinh). 2021 Jul 10;8(17):2101198. doi: 10.1002/advs.202101198 (PMC8425854; doi:10.1002/advs.202101198)
Supplement: Supplementary file 1 — Supporting Information [file ADVS-8-2101198-s002.pdf]

© 2021 Wiley-VCH GmbH

ADVANCED SCIENCE
Open Access

Supporting Information

for *Adv. Sci.*, DOI: 10.1002/advs.202101198

Remote Modular Electronics for Wireless Magnetic Devices

*Mustafa Boyvat and Metin Sitti**

Supplementary Information

Mustafa Boyvat and Metin Sitti

Supplementary Videos

Supplementary Video 1: Sequential remote assembly.

Supplementary Video 2: Use of external magnets for assembly.

Supplementary Video 3: Circular geometry formation by remote magnetic forces.

Supplementary Video 4: Circular geometry formation by mechanical stimulation in an all-modules-at-target scenario.

Supplementary Video 5: Circular geometry formation in continuous mechanical stimulation in a sequential and random delivery scenario.

Supplementary Video 6: Remote component replacement.

Supplementary Video 7: Remote geometry extension.

Supplementary Video 8: Assembly and powering of a robotic device with strong SMA actuator.

Supplementary Video 9: Heating distribution and temporal behavior.
